# An arch worth revisiting: a study on the feline humeral supracondylar foramen and its evolutionary significance

**DOI:** 10.1242/bio.060420

**Published:** 2024-06-25

**Authors:** Eimear Byrne, Robert D. Johnston, David Kilroy, Sourav Bhattacharjee

**Affiliations:** ^1^ School of Veterinary Medicine, University College Dublin, Belfield, Dublin, Ireland; ^2^ Trinity Centre for Biomedical Engineering, Trinity College Dublin, Dublin, Ireland; ^3^ Department of Mechanical, Manufacturing & Biomedical Engineering, School of Engineering, Trinity College Dublin, Dublin, Ireland

**Keywords:** Supracondylar foramen, Ligament of Struthers, Median nerve, Brachial artery, Coracobrachialis longus, Micro-computed tomography

## Abstract

The supracondylar foramen with a (seemingly) osseous peripheral arch noticed on the medio-distal feline humeri had remained disputed among anatomists. Some scholars have argued in favor of homology between this foramen and the supracondyloid foramen formed in presence of the ligament of Struthers in humans. Other theories include its presence as a retinaculum holding the median nerve and brachial artery to their anatomical position in a flexed elbow. Unfortunately, these theories lack investigative rigor. The emergence of non-invasive imaging modalities, such as micro-computed tomography, has enabled researchers to inspect the internal anatomy of bones without dismantling them. Thus, a micro-computed tomographic investigation was conducted on three feline (*Felis catus*) humeri specimens while the internal anatomy of the supracondylar foramina was examined. Unlike the humerus, the thin peripheral arch of the feline supracondylar foramen failed to elicit any osseous trabeculae or foci of calcification. While adhering to the humeral periosteum at its origin, the non-osseous arch, typical of a muscular tendon, attaches into the bony saddle related to the medial humeral epicondyle suggestive of a tendon or aponeurotic extension of a (vestigial) brachial muscle, with the coracobrachialis longus emerging to be the most likely candidate.

## Teaser

**Summary:** The feline supracondylar foramen was investigated using micro-computed tomography, and the novel insights assigned the peripheral arch a tendinous remnant of a digressing head of the coracobrachialis longus muscle.

## INTRODUCTION

**Fig. 1.**
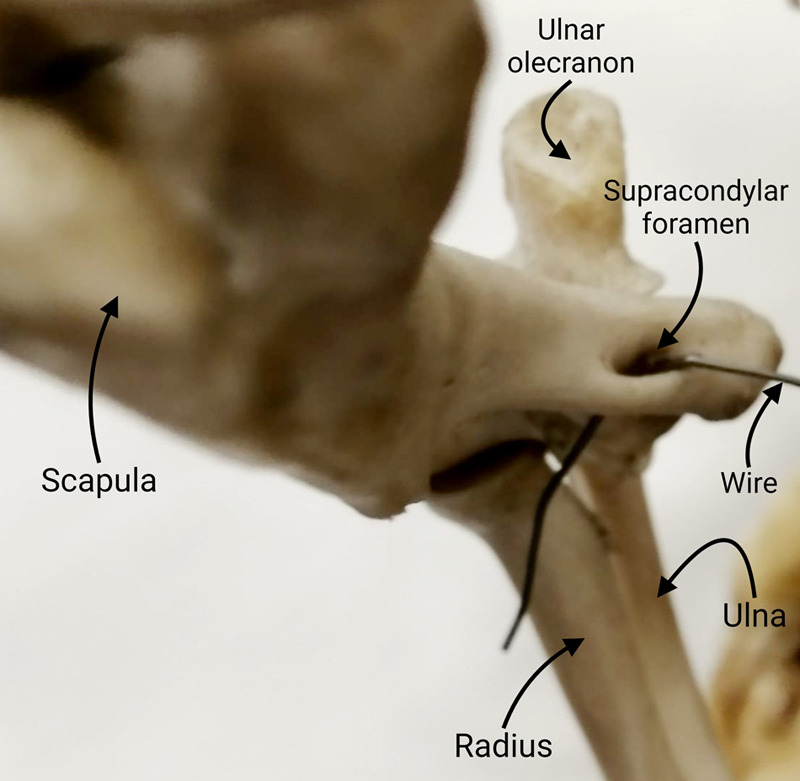
An oblique view of the elbow joint of a wallaby showing the articulating bones and the humeral supracondylar foramen (marked with a passage wire).

The humeral supracondylar foramen (Fig. 1), situated at the medio-distal aspect of the feline humeri, a few centimeters proximal to the medial epicondyle (Sánchez et al., 2013), has intrigued anatomists. Although not ubiquitous, the foramen is not rare and is also noted in climbing mammals (e.g. lemurs) and marsupials (e.g. wombats and koalas). The structures that pass through this foramen are the brachial artery and median nerve.

**Fig. 2.**
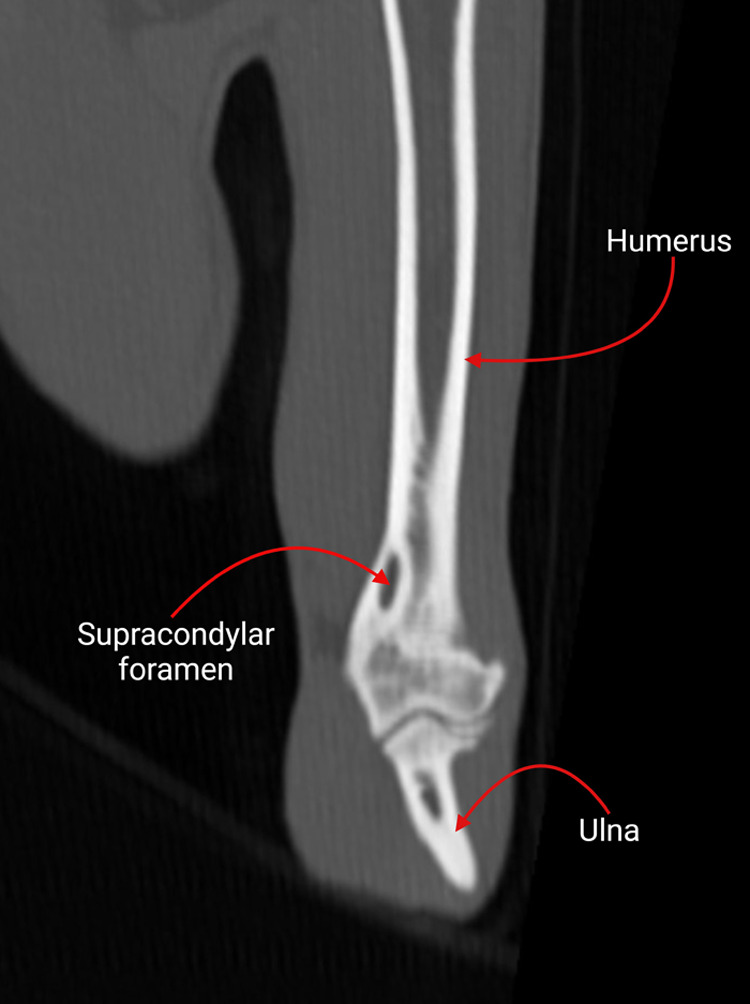
Computed tomographic image of a feline humerus showing the supracondylar foramen.

The function of this foramen remains obscure and, unfortunately, fosters speculation. Some have argued that it plays a protective role for the neurovascular bundle of the forelimb during contraction of the overlying flexor muscles (Huntington, 1918), external blows, and fluctuating pressure felt, especially during abduction when the superficial structures become more exposed, and are vulnerable to trauma. On the contrary, some researchers have opined that the foramen or its apparently rigid peripheral arch signifies the attachment site for the pronator teres muscle with a shifted site of origin (Ruge, 1884), although this thesis was later contested (Stromer, 1902). Additionally, it serves as a brace or retinaculum (Fig. 2) to prevent the median nerve from sagging forward into the angle of the elbow during flexion (Landry, 1958).

The filiform structures of the forelimb, including the vasculature, nerves, and muscle tendons, need to be held near the elbow joint during locomotion. The median nerve is especially vulnerable to such unwarranted mobility as it has no branches around the elbow to maintain its position. This exposes the neurovascular bundle to external trauma, given that it is only covered by overlying skin and a thin sheath of epitrochlearis muscle. Therefore, the theory of the supracondylar foramen acting as a retinaculum does bear merit. On the contrary, the brachial artery is relatively stable around the elbow joint due to its rich anastomoses and is not always entrapped by the foramen, further strengthening the argument in favor of a protective role.

The seemingly random occurrence of this foramen and its lack of consistency across species is also remarkable. It was suggested that the foramen is only present when the distal humeri are wide to allow a more direct route for the brachial artery and median nerve to access the antebrachium (Meckel, 1825). Conversely, a narrow humerus would obviate the need for such a foramen. However, this theory has lost traction as the foramen in feline species are known to bear narrow distal humeri.

**Fig. 3.**
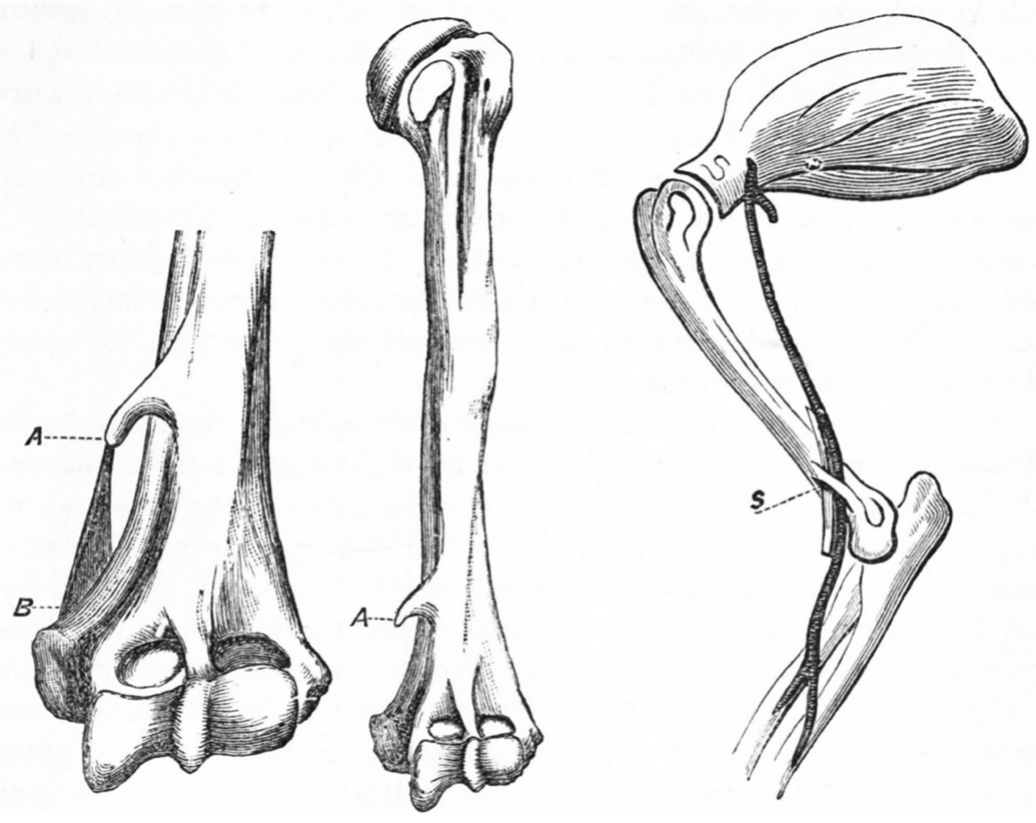
**The analogy between the feline supracondylar ligament and the ligament of Struthers in humans.** (A) The distally pointing supracondylar process proximal to the medial humeral epicondyle; (B) the ligament of Struthers as a fibrous band that, when present, forms the arch; (C) a medial view of the right feline humerus showing the supracondylar foramen with the median nerve and brachial artery passing through it. Modified and reproduced with permission from Struthers, 1873.

During an interspecies comparison, an interesting congruity was found between the feline species and its supposed homolog in ∼2% of humans. A bony spur, the supracondyloid process, is noted in the medio-distal aspect of human humeri a few centimeters proximal to the medial epicondyle (Natsis, 2008). It projects ∼2–20 mm from the medial humeral epicondyle and is connected to a thin ligamentous band of tissue, also known as the Struthers’ ligament, eponymized after the Scottish anatomist John Struthers (1823–1899), which extends from the supracondyloid process to the medial epicondyle forming an arch over the median nerve and, occasionally, the brachial artery (Struthers, 1873). The arch is known to cause pain and paresthesia in the distal arm by causing compression to the median nerve (supracondylar process syndrome, Fig. 3), often requiring surgical intervention (Opanova and Atkinson, 2014).

This study aimed to understand this peculiar supracondylar foramen, noticed in multiple species, including felines, and inspect it through an evolutionary lens. Thus, an in-depth understanding of the internal fabric of the anatomical structures contributing to this foramen was deemed necessary. Our driving hypothesis was that this foramen, especially its peripheral edge, is not osseous – as it deceptively appears – but the remnant of a muscular tendon. Hence, non-invasive imaging tools, such as micro-computed tomography (micro-CT), were prioritized, given their ability to demonstrate the anatomical structures without dismantling the specimens, such as bones (Hanson and Bagi, 2004).

Such emerging non-invasive tools provide imagery datasets that can further be analyzed with digital platforms, such as Fiji Is Just ImageJ (FIJI) software, an open-source image analysis platform that provides numerical readouts on their geometric, physical, and topographical attributes. The obtained data demonstrate the need to revisit such minute and peculiar anatomical structures, often considered serendipitous, that provide important insights into vertebrate evolution and, at times, warrant an upgrade or rectification of the current wisdom.

## RESULTS

### Internal features of the foramen

**Fig. 4.**
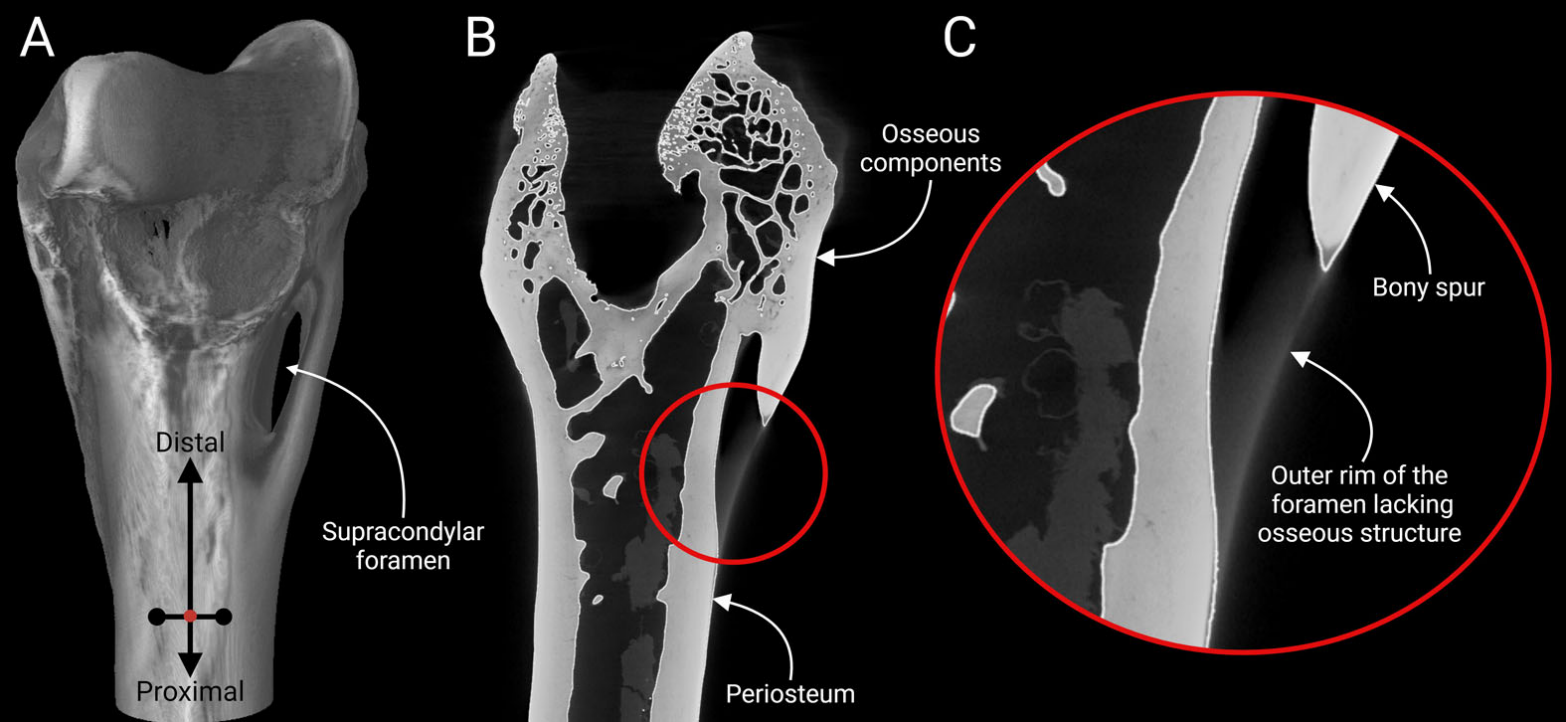
**The distal feline humerus was viewed under micro-CT imaging.** (A) Digital 3D reconstruction of the humerus; (B) the internal tissue fabric demonstrate a periosteum and trabeculae with a bony spur attached to a less rigid tissue; (C) a closer look into the bony spur with attached tissue band in a region of interest encircled within a red circle.

The micro-CT investigation acquired high-resolution scans through the feline (*Felis catus*) humeri at different levels, and the *z*-stacks were then combined to develop a digital 3D rendition of the distal feline humerus in VG studio (https://www.volumegraphics.com/en/products/vgstudio.html) with a clear demonstration of its anatomical landmarks, including the trochlea and the supracondylar foramen (Fig. 4A; Movies S1 and S2). The micro-CT data obtained from the other two feline humeri (Fig. S1) corroborated with the representative specimen documented here. Internally, the bony trabeculae could be noticed inside the distal humerus, while the humeral shaft was wrapped in a periosteum (Fig. 4B). However, the typical osseous architecture was missing at the outer rim/arch of the foramen attached seamlessly to the humeral periosteum, and a shadow of less rigid, non-osseous tissue was detected (Fig. 4B). This tissue, without hard evidence of calcification, was attached to a bony spur related to the medial condyle (Fig. 4C).

### Osseous trabeculae

**Fig. 5.**
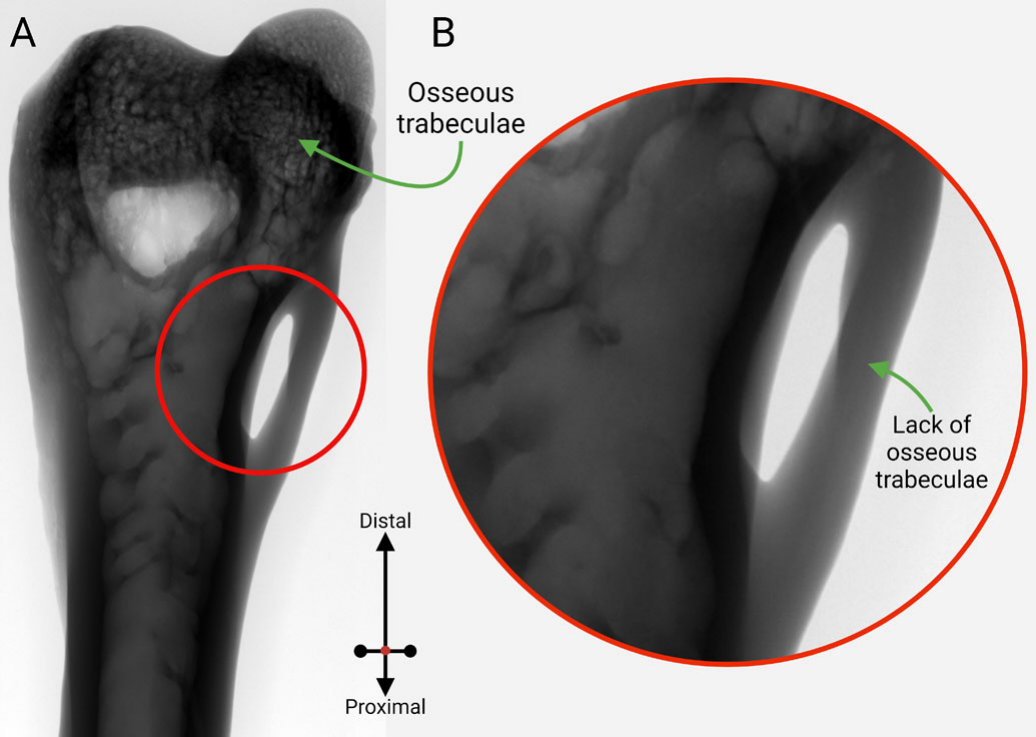
**The scout view from a micro-CT scan demonstrated the osseous trabecular trails inside a distal feline humerus.** (A) Extensive trabeculae, characteristic of long bones, were noticed inside the humerus, including its shaft, condyles, and articulating surface, whereas no such trails were detected in the soft unossified tissue forming the rigid outer arch of the foramen; (B) a closer look into the region of interest on the supracondylar foramen marked as a red circle.

The micro-CT scan also revealed trails of extensive osseous trabeculae within the feline humerus, including its shaft, condyles, and articulating areas, whereas no such trabeculae could be identified in the unossified softer tissue mass connected to the distal humerus (Fig. 5).

### 3D surface projection in FIJI

**Fig. 6.**
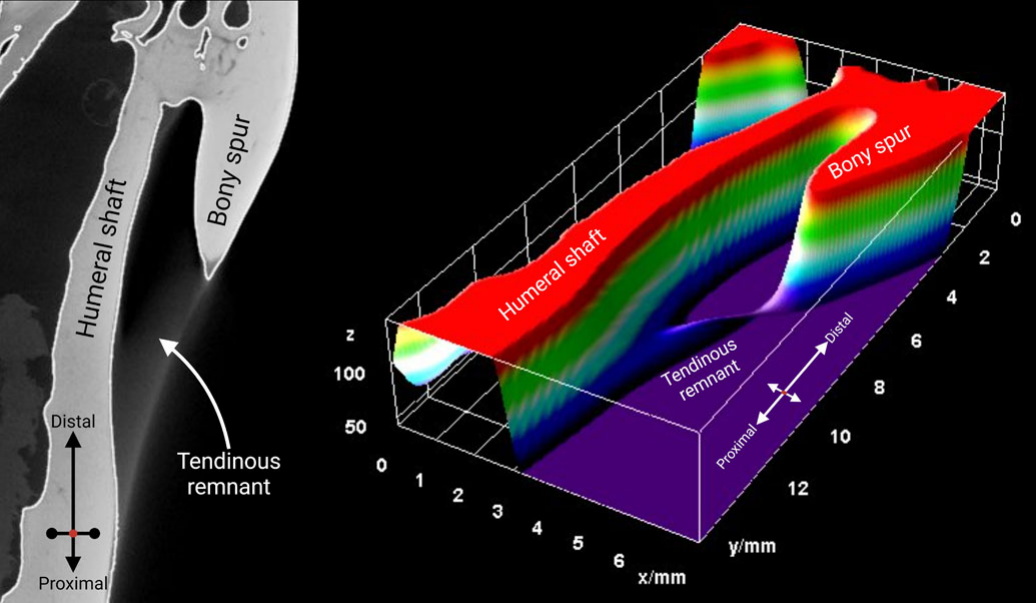
**The 3D surface projection of a feline humeral supracondylar foramen rendered in FIJI software.** The bony spur was attached to the humeral shaft, while the tendinous remnant, acting as the outer rim of the foramen, lacked an osseous fabric and was attached to the humeral shaft. The scale of the grids is included in the figure.

The 3D surface projection by FIJI categorically demonstrated the presence of a bony shaft attached to the edge of the humeral shaft. On the contrary, the tendinous remnant could be demarcated from the neighboring bony structures, including the humerus (Fig. 6). The 3D rendition also demonstrated that the tendinous remnant was attached to the humeral periphery.

## DISCUSSION

The micro-CT investigation divulged granular details of the internal anatomy of the feline distal humeri. Moreover, with the help of *in silico* tools, such as FIJI, a realistic digital reconstruction of the feline humeri with crisp resolution was achieved. The availability of such sophisticated imaging platforms harbors the potential to revolutionize anatomy pedagogy and research. The micro-CT data demonstrated the presence of an unossified band of tissue at the outer periphery of the foramen, and to the best of our knowledge, this is the first systematic study on feline supracondylar foramen based on internal anatomy by emerging imaging tools like micro-CT. There was speculation made in the past that it was a homolog of the supracondylar process attached to the ligament of Struthers (Kessel and Rang, 1966). Intriguingly, in his 1873 paper on the hereditary and humeral supracondyloid process in humans, John Struthers stated that this process would “be found to occur as a variety also among some of the higher animals in which it does not exist normally, were they examined as often and as carefully as the human body is”.

Our data support this analogy, and an obvious similarity in external appearances further strengthens the thesis. As confirmed by micro-CT, a relatively softer tissue band is characteristic of tendinous presence at a muscular insertion site, for example, at the insertion of the Achilles tendon on the calcaneal tuberosity (Pasetto et al., 2018). Under micro-CT, the softer tissues typically project hazy shadows due to deficient ossification, while our data supported the notion that the supracondylar foramen's arch is a tendinous remnant of a (vestigial) muscle. Furthermore, the extent of this soft tissue band was entirely within the distal humerus and, unlike a ligament that typically connects two bones, did not link the humerus to any other bone. This leaves a muscular tendinous vestige as the only feasible option to explain this arch devoid of ossification.

Considering the trajectory of this tendon vestige, especially its attachments and directionality, this vestigial muscle is brachial. This automatically excludes the supracondylar and pronator teres muscles, or in the case of cats, the epitrochlearis muscle, based on the argument that these are primarily forearm (antebrachial) muscles. Tendons of other muscles that traverse near the elbow joint, such as the brachialis and triceps brachii (especially its caput mediale accessorium component), also do not fit the anatomy of this structure, given both these muscles (de Souza Junior et al., 2018; Dunn et al., 2022; Ercoli et al., 2015; Fisher et al., 2009; Julik et al., 2012) originate (mostly) in scapula and insert into the proximal part of the radius and ulna (brachialis) or ulnar olecranon (triceps brachii). On the contrary, this tendon vestige is limited within the distal humerus and does not extend further. Thus, from a reductionist approach, any case for brachialis or triceps brachii may be negated.

**Fig. 7.**
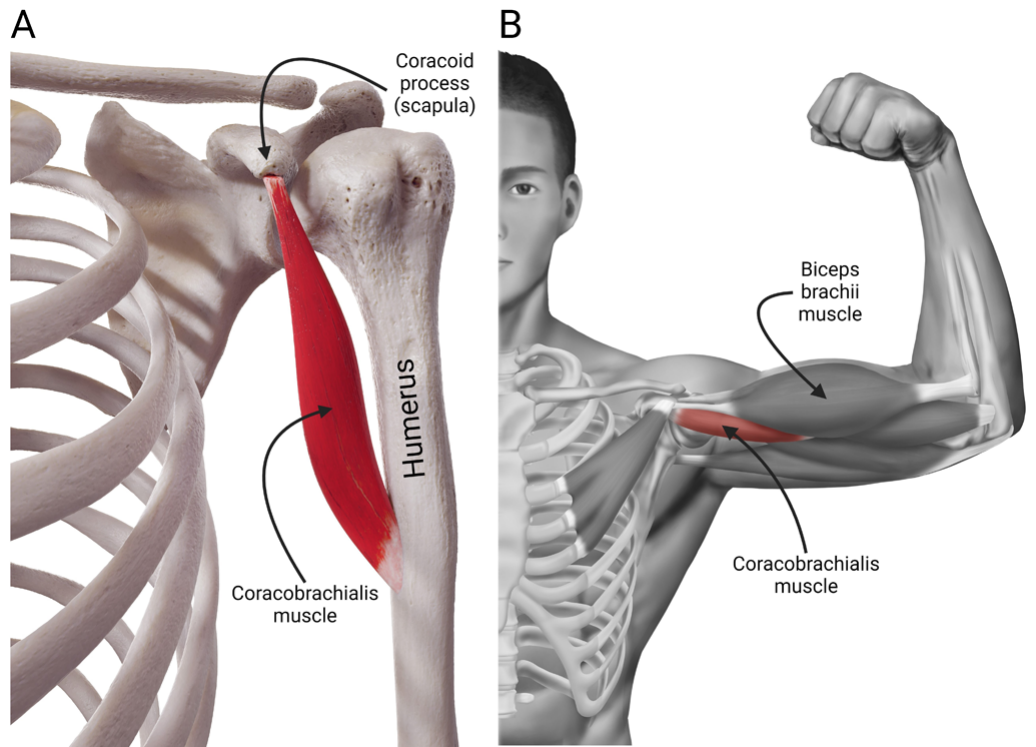
The anteroposterior views of the coracobrachialis muscle in humans when the arm is in (A) anatomical, and (B) abducted positions.

The coracobrachialis muscle is the only feasible brachial muscle contributing to this bony arch around the supracondylar foramen (Fig. 7). While taking its primary origin from the scapular coracoid process, the coracobrachialis muscle is inserted into the medial third of the humerus as the only surviving adductor muscle of the arm in humans. In many species, including reptiles and amphibians, the muscle has three heads, for example, coracobrachialis longus, also known as Wood's muscle (Wood, 1867) – a morphologically variable head known to extend distally to the median nerve and brachial artery while staying related to the humeral shaft; coracobrachialis medius, which is inserted into the humeral shaft distal to the insertion of latissimus dorsi muscle; and coracobrachialis brevis, which inserts into the medial humeral shaft proximal to the insertion of latissimus dorsi (Guha et al., 2010). However, the muscle – with all its heads – can be identified in many non-human species (Fig. S2).

In humans, the coracobrachialis muscle has two heads surrounding the musculocutaneous nerve (Vélez-García et al., 2023; Zielinska et al., 2022), and occasional nerve compression gives rise to clinical symptoms requiring surgical intervention. The coracobrachialis longus – although digressed almost entirely now – is known to give rise to a tendinous insertion on the humerus with an aponeurotic extension that reaches the distal end of the supracondylar ridge with ligamentous extension to the humeral inner condyle. Our data indicated that the unossified and softer tissue connected to the characteristic distal humerus in humans might be an aponeurotic extension or its remnant.

In the past, there had been plenty of confusion with the supracondylar foramen, ligament of Struthers, and the entepicondylar foramen – a foramen situated at the distal humerus of some amniotes – and noticed in fossils of archaic dinosaurs (Figueirido, 2020). Unfortunately, these terms have often been used injudiciously, and wild speculations made from purely external appearances further complicate comprehension. This study has established that the supracondylar foramen, noticed in a feline skeleton, is a remnant from a vestigial arm muscle, with the coracobrachialis longus emerging as the most suitable candidate. However, an antebrachial muscle cannot be excluded completely. It is difficult to speculate which muscle it can be, although a proximally displaced origin of pronator teres emerges as an option, given that such a displacement might enjoy an evolutionary favor by providing improved grip.

As a historical note, even Charles Darwin was curious about such an opening near the medial humeral epicondyle and contacted John Struthers. In his book The ‘Origin of Man and Selection in Relation to Sex’ (1871), Darwin found it rather peculiar that humans elicit the Struthers’ ligament, which is absent in higher Quadrumana and thus may account for an evolutionary inversion where anatomical structures, rather than diversifying over evolution – with older structures replaced with new ones – reverted to their archaic forms. The tetrapod evolution transitioned from water to land as their primary habitat during the Devonian period (419.2–358.9 million years ago), and a fin-to-limb evolution of the forearm ensued with considerable modulation of the distal humerus. In sarcopterygian fishes, the median nerve and brachial artery run along a channel situated within a ventral crest on the humeral shaft before entering the forearm via a foramen, which may be an archaic form of the entepicondylar foramen. However, with evolution, the course of the median nerve and brachial artery gradually changed, passing from the dorsal to the ventral side of the humerus (Landry, 1958). It would be fascinating to compare the entepicondylar foramina noticed in dinosaurs with feline supracondylar foramina, and the probability of them being the same, or at least homologous, is worth investigating.

This alteration in trajectories was perhaps accelerated by the emergence of brachiation that required restructuring of the brachial plexus, with a conversion of its directionality from an anteroposterior to a proximodistal fashion (Hirasawa and Kuratani, 2018) along with a drop and simultaneous torsion of the distal humerus resulting in depression. During the late Devonian and Carboniferous period (359.2–299 million years ago), the entepicondylar foramen appeared to provide the median nerve and brachial artery a corridor to the forearm, ensuring a sustained – and perhaps an augmented – flow of blood while making concessions for a more extensive innervation plexus to deal with an increasing workload and complex biomechanics (Smithson and Clack, 2018).

Some have argued that the necessity of the entepicondylar foramen gradually diminished, with the ungulates mostly moving their forelimbs in one plane without humeral abduction. On the contrary, perhaps paradoxically, in humans, it has disappeared due to a much larger range of abduction (Landry, 1958). It is difficult to speculate on the evolutionary discourse of its appearance and subsequent disappearance – with occasional atavisms, such as the ligament of Struthers – in addition to its incredible variation noted even within the same species, family, or genus.

Weighing the data, it is prudent to accept that our understanding of the foramen near the medial humeral epicondyle needs a further boost. The complex genetic interactions and expressions should also be factored in. The data obtained in this study have elucidated that, at least in cats, the outer arch of the supracondylar foramen is a remnant from a vestigial muscle, which (possibly) is the coracobrachialis longus. However, the anatomy of the supracondylar process connected to the ligament of Struthers, or the entepicondylar foramen, remains far from settled. One of the reasons behind this confusion is that these three foramina are known for entrapping the same forelimb structures: the median nerve and brachial artery. Moreover, their proximity to the medial epicondyle and an undeniable external similarity leaves ample room for interpretation. That perhaps reiterates the primordial caution while dealing with anatomical specimens: visual similarity does not guarantee equality.

Given the extensive adaptation that the distal humerus underwent during its evolutionary journey, especially in its relations to the median nerve and brachial artery, any arch – ossified or not – near the medial humeral epicondyle will invariably engage these two structures, with full or partial entrapment. Further follow-up investigations might reveal fascinating insights on entepicondylar foramen, indicating whether it is a mere homolog of these two structures mentioned earlier or stands alone as an independent anatomical landmark. Any such investigation on the entepicondylar foramen should also embrace old, fossilized specimens from extinct forms of vertebrates, dinosaurs included, as that may help raytracing its evolutionary trajectory and ambit.

Unfortunately, the success of micro-CT depends on the specimen material. Its quality, especially in fossilized specimens, will require plenty of optimization and data processing, with varying reliability of the extracted output, which can be challenging. Further limitations include the availability of an adequate number of specimens, preferably from multiple species, for comparative assessment. Furthermore, morphometric analysis on these specimens using digital platforms requires skill development and, at times, access to computational facilities, including data storage servers, when dealing with larger datasets – which, depending on circumstances, may not be instantly available.

## MATERIALS AND METHODS

### Feline humeri

Three feline (domestic cat, *F. catus*) humeri specimens were collected from the anatomical specimen repository of the University College Dublin School of Veterinary Medicine and subjected to further micro-CT analyses. The study received approval from the Animal Research Ethics Committee of University College Dublin, with an exemption code of AREC-20-21-Kilroy.

### Micro-CT data acquisition

The micro-CT on feline humeri was performed using a Nikon XT H 225 micro-CT scanner. Table 1 provides further specifics on the micro-CT acquisition. Ring correction and two averages were also performed to remove artifacts from the final images and ensure robust data collection. The acquired images were exported in JPEG format for further 3D surface projection in FIJI.

**Table 1.**
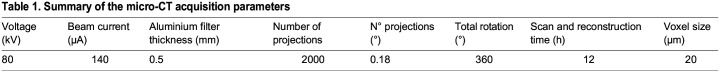
Summary of the micro-CT acquisition parameters

### 3D surface projection in FIJI

FIJI is an open-source software freely available for download (https://fiji.sc/) and used for image computing, 3D rendition of (anatomical) structures, segmentation, and calculation of various 2D and 3D structural attributes for plotting and visualization. It is compatible with popular operating systems, including Windows, Linux, and macOS, and offers a user-friendly, intuitive interface with a further opportunity to increase the depth of analyses by installing tailor-made plugins.

For a 3D surface projection of the supracondylar foramen, the region of interest was cropped out of the micro-CT image, followed by processing by the workflow: FIJI→Plugins→3D→Interactive 3D Surface Plot. The (interactive) 3D surface plot was rendered in Thermal LUT (lookup table) under the following settings: Grid size: 256; Smoothing:12; Perspective: 0.2; Lighting: 0.69; z-scale: 0.22; Max: 55%; Min: 17%. The image scale was set using the following widgets: FIJI→Analyze→Set Scale (Fig. S3).

## Supplementary Material

10.1242/biolopen.060420_sup1 Supplementary information
