# Supplementary material for: An arch worth revisiting: a study on the feline humeral supracondylar foramen and its evolutionary significance
Source: Biol Open. 2024 Jun 25;13(6):bio060420. doi: 10.1242/bio.060420 (PMC11225584; doi:10.1242/bio.060420)
Supplement: Supplementary information [file biolopen-13-060420-s1.pdf]

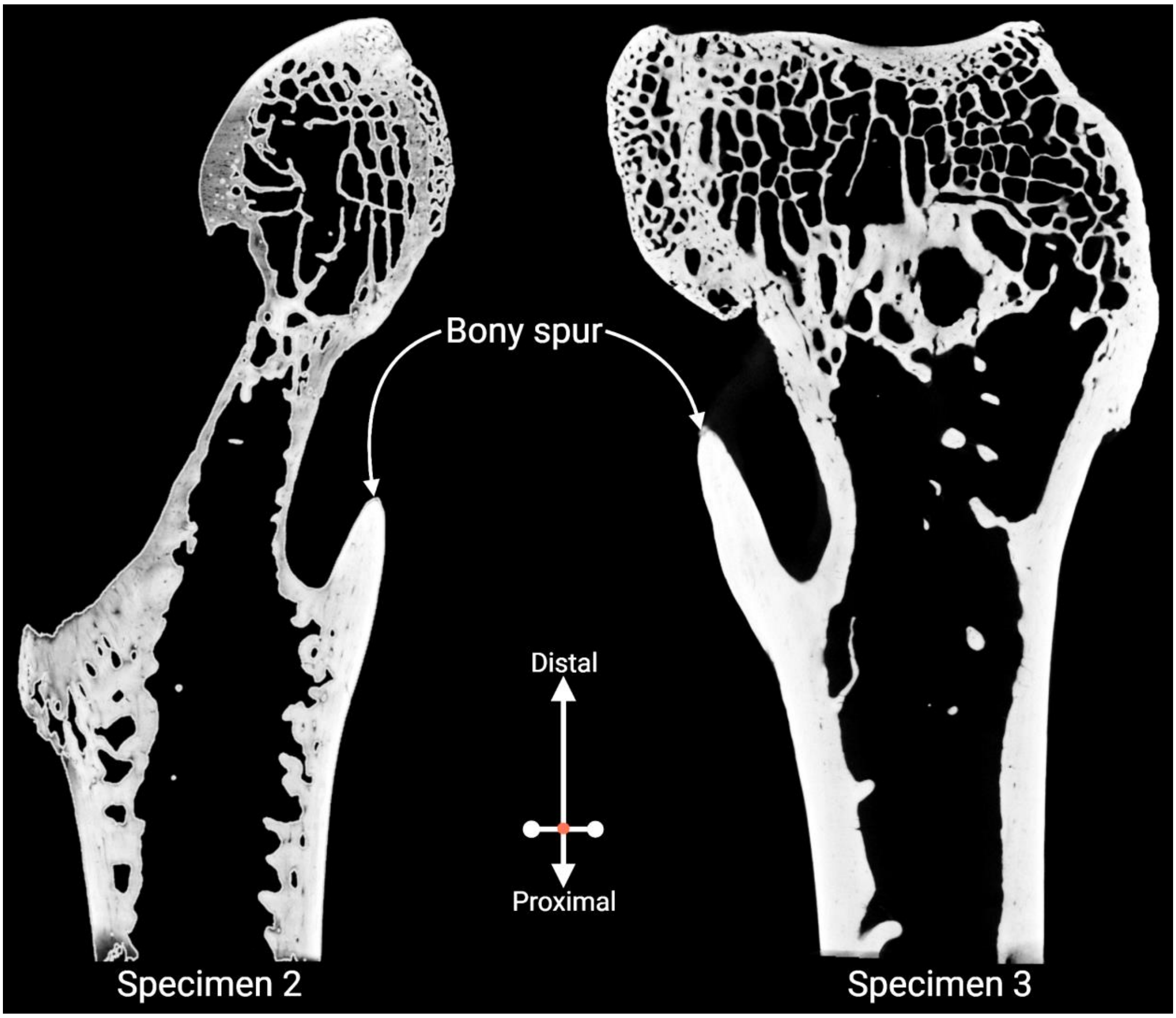


**Fig. S1.** The micro-CT data on the remaining two feline humeri specimens demonstrate the presence of bony spurs similar to the specimen reported in the main manuscript. A proximodistal arrow is embedded within the figure.

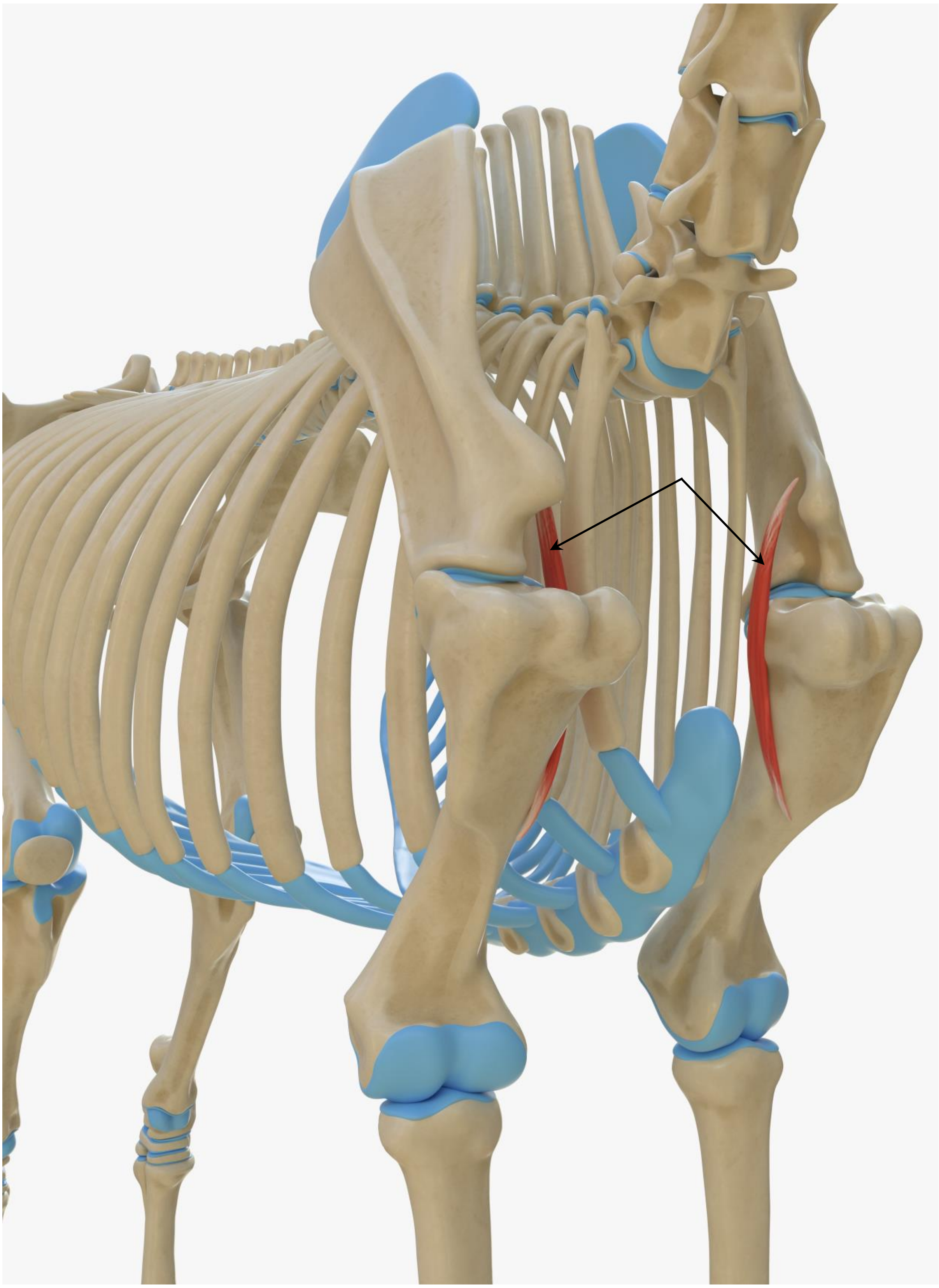

**Fig. S2.** Coracobrachialis muscle noticed in other non-human species.

The coracobrachialis muscle (marked with arrows) in horses.

Biology Open • Supplementary information

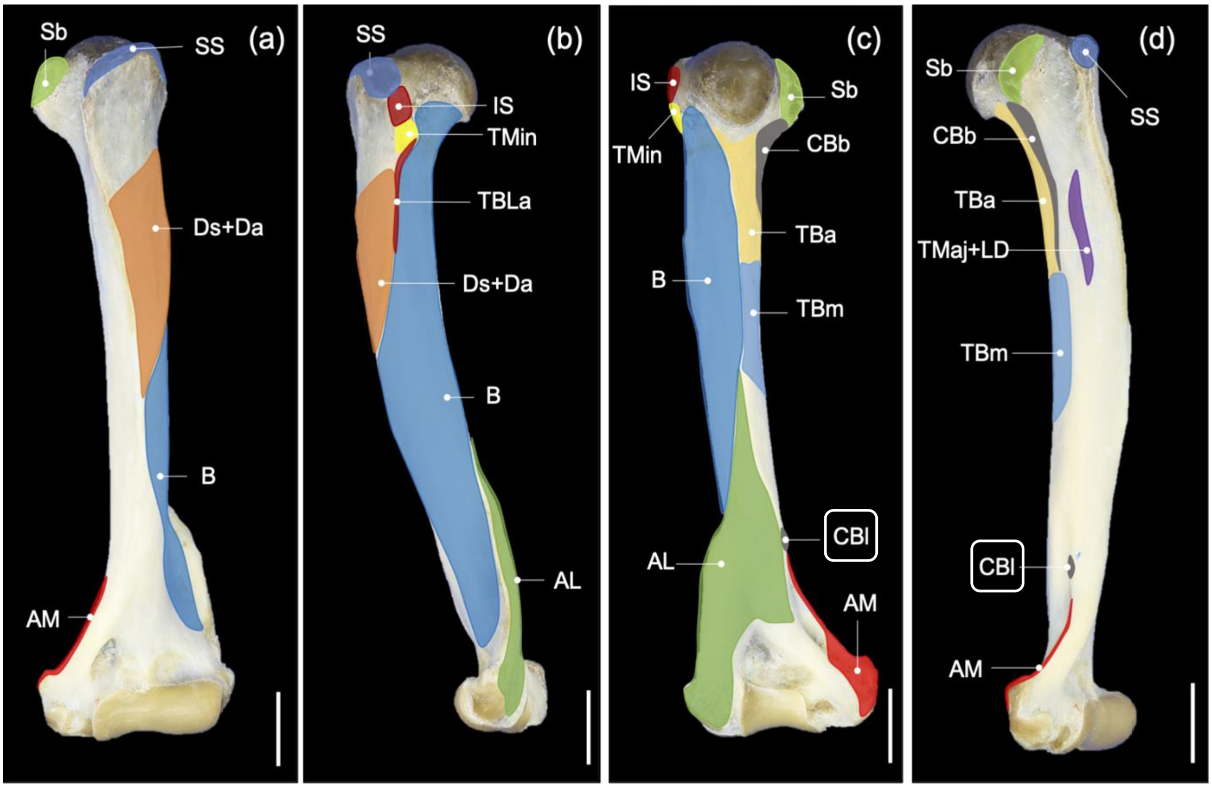


The muscular attachment mapping of the shoulder and brachial muscles in the left humerus of tropical rainforest mammal Kinkajou (*Potos flavus*). Panels (a)–(d) show the cranial, lateral, caudal, and medial views, respectively. The coracobrachialis longus muscle is abbreviated as "CBl" and marked within a box in Panels (c) and (d). Abbreviations of other muscles: AL, anconeus lateralis; AM, anconeus medialis; B, brachialis; CBb, coracobrachialis brevis; Da, deltoideus pars acromialis; Ds, deltoideus pars scapularis; IS, infraspinatus; LD, latissimus dorsi; Sb, subscapularis, SS, supraspinatus; TBLa, triceps brachii caput laterale; TBm, caput mediale; TBa, caput accessorium; TMaj, teres major; TMin, teres minor. Scale bars 10 mm. Reproduced from Vélez-García et al., 2023, as freely available open-access material under the terms of the Creative Commons Attribution License (CC BY 4.0).

Biology Open • Supplementary information

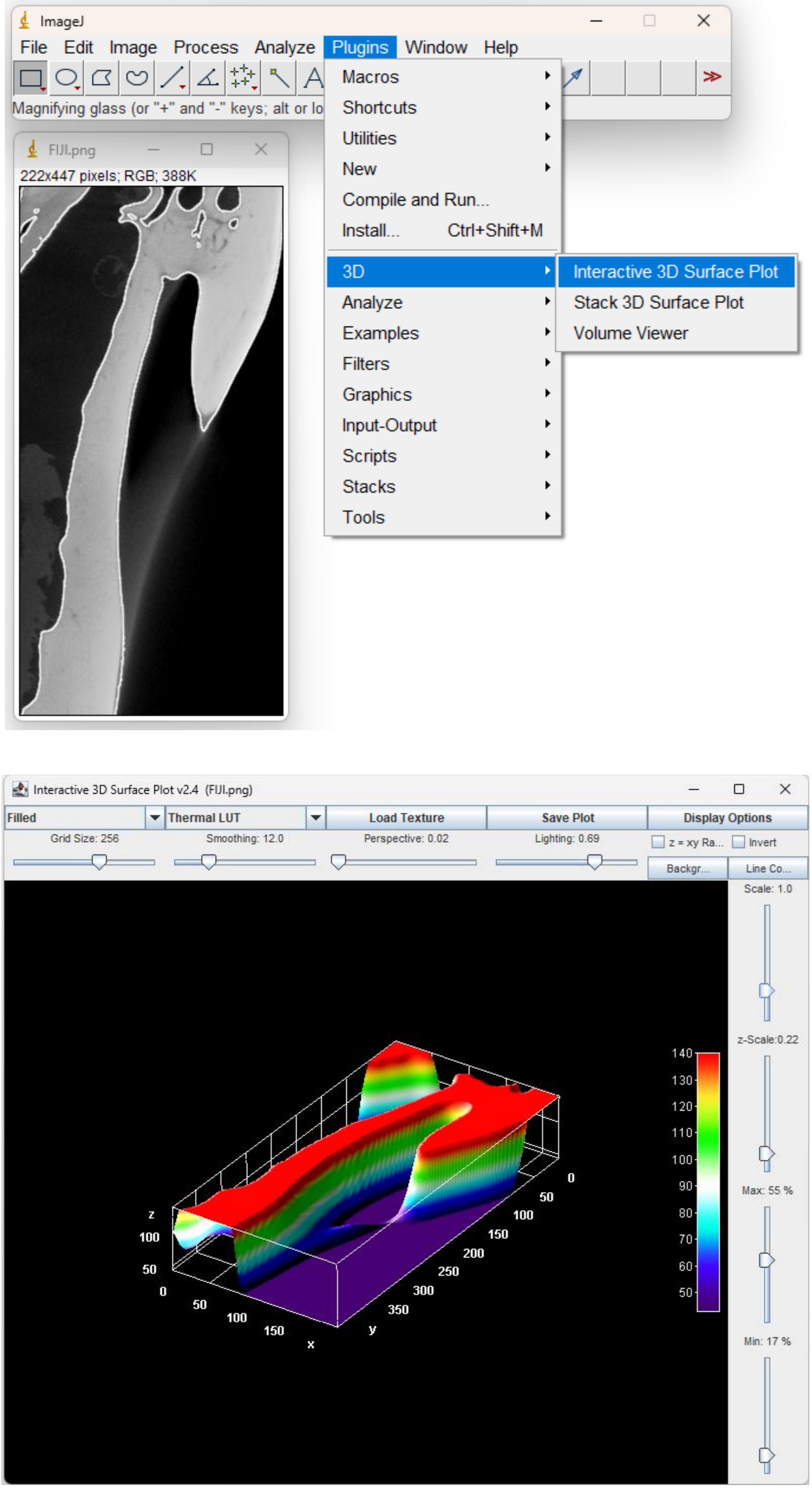


**Fig S3.** The workflow of creating the interactive 3D surface projection.

Biology Open • Supplementary information

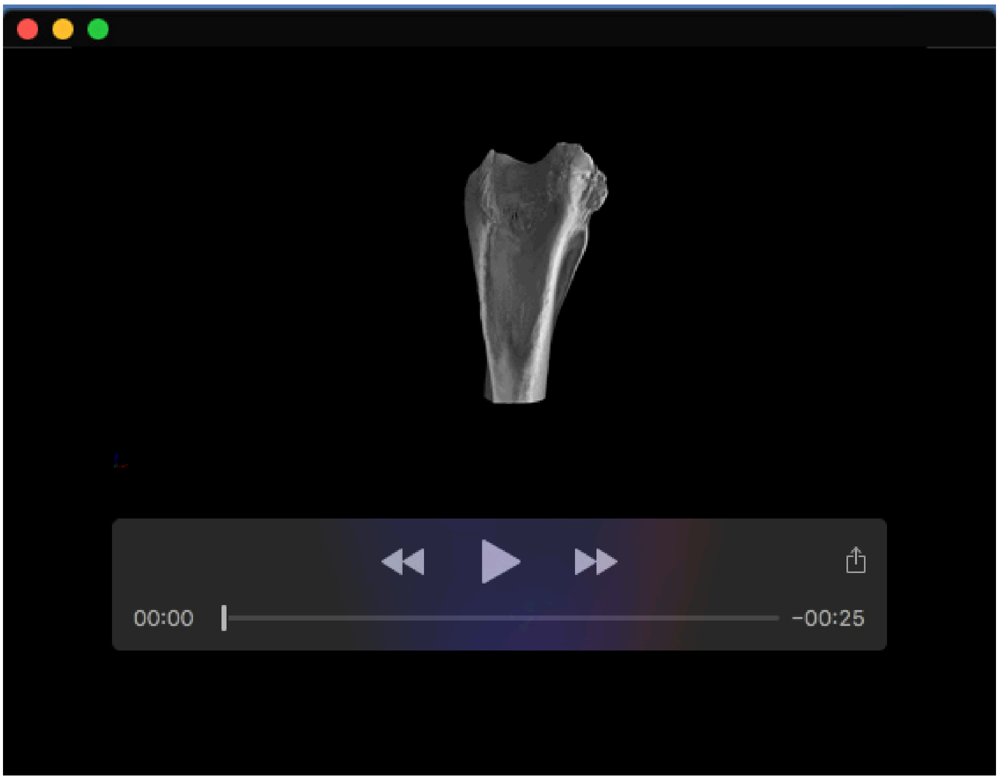


**Movie 1.**

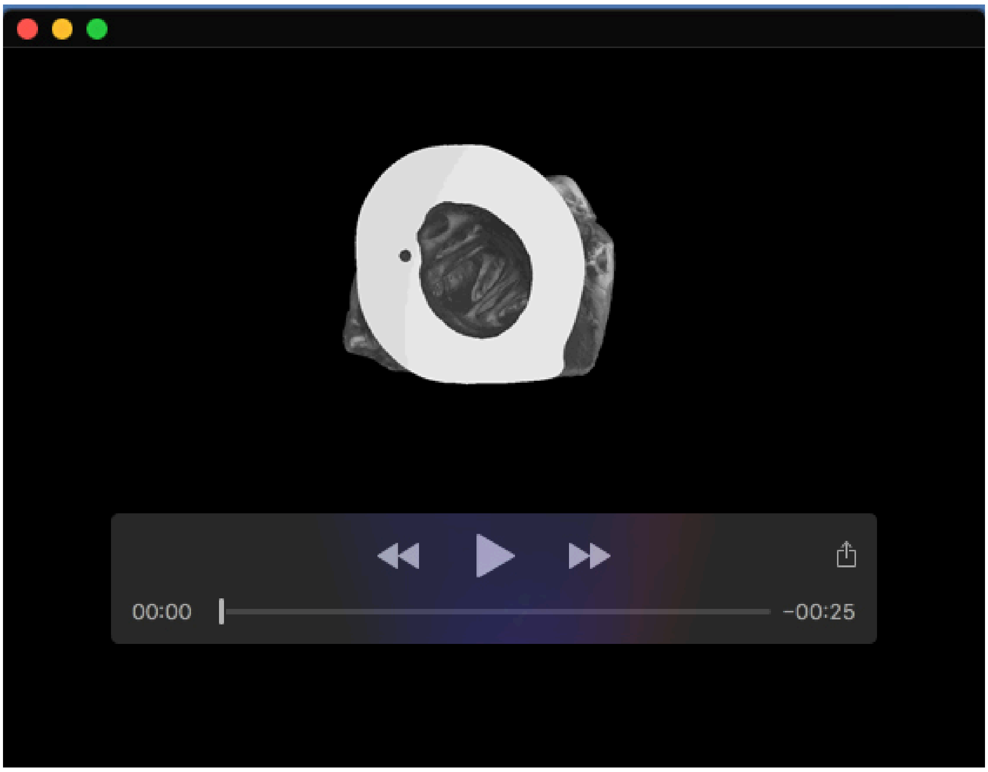


**Movie 2.**
